# The effect of topical atropine on the choroidal thickness of healthy children

**DOI:** 10.1038/srep34936

**Published:** 2016-10-07

**Authors:** Zhengwei Zhang, Yuanting Zhou, Zhifang Xie, Tiantian Chen, Yan Gu, Shui Lu, Zhifeng Wu

**Affiliations:** 1 Department of Ophthalmology, Nanjing Medical University Affiliated Wuxi Second Hospital, Wuxi, Jiangsu Province, P. R.China

## Abstract

The purpose of the current study was to investigate the effect of topical atropine on choroidal thickness using spectral-domain optical coherence tomography. A total of 30 healthy eyes from 30 children were analyzed in this study. A single drop of 1% atropine gel was administered twice daily for a week. Choroidal thickness (CT) was measured using SD-OCT, and changes in CT before and after administration of the eye drops were analyzed at the subfovea and at 1.0-mm intervals (up to 3.0 mm) from the fovea at superior, inferior, nasal, and temporal locations. Pre- and post-cycloplegic axial length (AL) was also measured using the IOLMaster. We observed that administration of 1% atropine gel led to a significant increase in the choroidal thickness under the fovea and at all intervals from the fovea. The greatest change in CT was observed in the inferior meridian, while the nasal meridian exhibited the least change. AL did not significantly differ before and after cycloplegia, and there was no significant correlation between the changes in AL and subfoveal CT. It was concluded that administration of 1% atropine gel can significantly increase CT in the eyes of young Chinese children, albeit with different magnitude at different locations.

The ocular choroid is an extensively vascularized tissue situated between the sclera and Bruch’s membrane. The major role of the choroid is to supply oxygen and nutrients to the outer retina^1^. Evidence from animal studies have reported that the choroid can rapidly change its thickness in response to a variety of stimuli such as myopic or hyperopic defocus in order to move the retina towards the plane of focus.

In addition, previous studies have revealed that there is a rich autonomic vasoactive nerve supply to the choroid that involves the activation of adrenergic and muscarinic receptors^2^. Therefore, it is reasonable to speculate that topical adrenergic and anticholinergic agents could exert an effect on the choroid. A number of animal studies have shown that non-selective, partially selective or highly selective muscarinic antagonists (i.e., atropine, pirenzepine, himbaicine, MT3, MT7) can significantly influence choroidal thickness and ocular growth changes^3^,^4^,^5^.

In humans, there are some reports that highlight the effect of topical anticholinergic or adrenergic agents on choroidal thickness with varied or even contradictory findings. For example, some studies have reported absence of any influence of these agents on the choroidal thickness^6^,^7^,^8^, while some authors have observed an increase^9^ or a decrease^10^,^11^ in the choroidal thickness, even when the same drugs were used (Table 1). We speculate that such discrepancies might arise from the differences in the methods of drug instillation, race of participants, and distribution rate of mydriatic agents to the posterior.

Atropine now is considered as a most potent drug to control the progression of myopia in children, with strong supporting evidence from well-conducted clinical trials^12^,^13^. However, the exact site and mechanism of action of atropine in slowing myopia progression is still insufficiently understood^14^,^15^. In a longitudinal study, Read and associates^16^ reported that a significant increase in subfoveal choroidal thickness of myopic and nonmyopic children was observed over 18-month follow-up, and children showing faster axial eye growth exhibited significantly less choroidal thickening over time compared with children showing slower axial eye growth. The results suggested that there may be a potential role for the thicker choroid in the mechanisms inhibiting eye growth in childhood. In this respect, if the choroid of children is thickened by drugs (i.e. atropine), which might be a part of mechanism to slow the progression of myopia.

By far, however, there is no published evidence of the effect of topical atropine administration on the choroidal thickness of children *in vivo* measured by SD-OCT equipments. Therefore, in the present study, we wanted to explore the effect of a long acting anticholinergic drug i.e. atropine (1%) on the choroidal thickness in Chinese children within the age group of 5 to 10 years.

## Results

In the present study we examined 30 healthy eyes of 30 subjects (including 14 boys) satisfying the inclusion criteria. The subjects were in the age group 5-10 years with a mean age of 7.47 ± 1.16 years. The mean spherical equivalent after 1-week of cycloplegia was -0.38 ± 2.16 D (from −3.50 D to +4.63 D).

**Figure 1:**
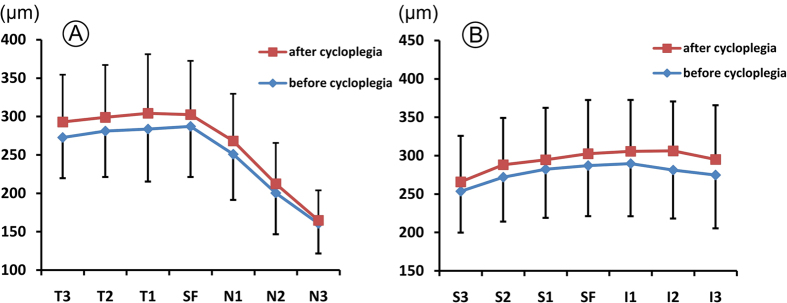
The changes of choroidal thickness before and after 1-week administration of 1% atropine gel. (**A**) T3, 3 mm temporal to the fovea; T2, 2 mm temporal to the fovea; T1, 1 mm temporal to the fovea; SF, subfovea; N3, 3 mm nasal to the fovea; N2, 2 mm nasal to the fovea; N1, 1 mm nasal to the fovea. (**B**) S3, 3 mm superior to the fovea; S2, 2 mm superior to the fovea; S1, 1 mm superior to the fovea; SF, subfovea; I3, 3 mm inferior to the fovea; I2, 2 mm inferior to the fovea; I1, 1 mm inferior to the fovea.

Choroidal thickness measured at the fovea and at 1.0-mm intervals (up to 3.0 mm) from the fovea at nasal, temporal, superior, and inferior locations for both groups are summarized in Table 2. Following administration of 1% atropine gel, choroid thickened significantly under the fovea (i.e. from 287.03 ± 65.76 μm to 302.52 ± 69.94 μm, *P* < 0.001) and at all intervals from the fovea. However, the magnitude of change in choroidal thickness varied with the location. The greatest change in choroidal thickness was observed in the inferior meridian (20.44 ± 30.21 μm, the average change of the three measurement locations in the inferior meridian, the same below), while the nasal meridian exhibited the least change (11.19 ± 15.54 μm) (*P* = 0.007, Fig. 1). In addition, the change of choroidal thickness in the temporal meridian (19.52 ± 24.06 μm) significantly thickened than that of in the nasal meridian (*P* = 0.016), and the change of choroidal thickness in the inferior meridian slightly but significantly thickened than that of in the superior meridian (13.66 ± 21.71 μm) (*P* = 0.048).

Axial length did not differ significantly before (23.40 ± 0.98 mm) and after (23.39 ± 0.98 mm) cycloplegia with a mean difference of −8.33 ± 46.32 μm (*P* = 0.333). Plus, there was no significant correlation between the changes in axial length and subfoveal choroidal thickness (*r* = −0.186, *P* = 0.324).

**Figure 2:**
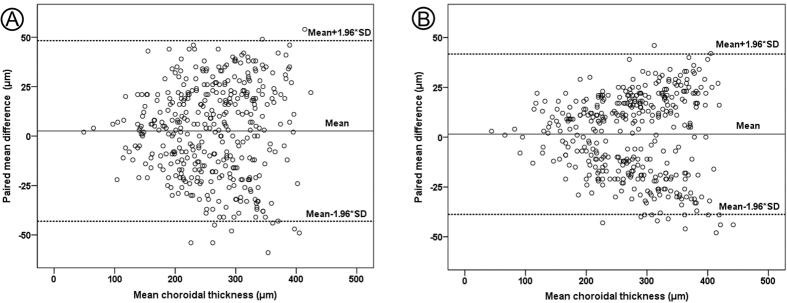
Bland-Altman plot analyses. Bland-Altman plot of interobservers agreement on choroidal thickness measurements for all locations between the two observers before (**A**) and after 1-week administration of 1% atropine gel (**B**). Solid line indicates the average mean difference, whereas dotted lines delineate 95% limits of agreement (1.96 *SD). There was no specific trend to cause the difference between the two observers.

There was excellent agreement between the two independent observers, with an ICC of 0.952 and 0.955 before and after cycloplegia, respectively (Table 3). Bland-Altman plot of difference against mean choroidal thickness showed no significant change in variability for the range of choroidal thickness, and a very few observations were outside 95% limits of agreement (LOA) both before and after cycloplegia (Fig. 2). Specifically, the Bland-Altman analysis indicated that the 95% LOA between the two observers ranged from −43.09 μm to 48.31 μm (mean, 2.61 μm) and −38.11 μm to 41.71 μm (mean, 1.47 μm) for chorodial thickness of all locations of measurements before and after cycloplegia, respectively (Table 3). Plus, the inter-observer repeatability coefficients were 46.58 μm (95% confidence interval: 45.55 μm to 47.32 μm) and 40.96 μm (95% confidence interval: 39.58 μm to 41.14 μm) for chorodial thickness of all locations of measurements before and after cycloplegia, respectively (Table 3).

## Discussion

Similar to the iris, which is also a part of the uvea, the choroid may also likely display certain changes following the use of a mydriatic agent. In the present study, we found that the use of topical 1% atropine gel administration for a week significantly increased the choroidal thickness under the fovea and at all parafoveal locations. Our findings are consistent with the results of Sander *et al*., wherein 2% homatropine hydrobromide was used^9^. Notably, we did not observe a statistically significant change in the axial length (−8.33 ± 46.32 μm) despite significant subfoveal choroidal thickness (15.48 ± 16.13 μm) as a result of atropine-induced cycloplegia. Plus, we did not discover a significant relationship between the changes in subfoveal choroidal thickness and axial length, suggesting that choroidal thickening could not significantly influence the axial length obtained by IOLMaster. We speculated that the lack of correlation between the change of axial length and subfoveal choroidal thickness may be attributed to the relatively poor resolution of IOLMaster (10 μm) when compared with SD-OCT (5 μm). It was noteworthy that the range of standard deviation of the change of the axial length was relatively wide.

In the present study, although choroidal thickness thickened significantly at all the measured locations, the magnitude of change was not identical. Maximal change in choroidal thickness was observed in the inferior meridian (20.44 ± 30.21 μm), while the location 3.0 mm nasal to the fovea exhibited the least change (4.17 ± 10.49 μm) in choroidal thickness which may be attributed to the limited space around the optic head (Fig. 1; Table 2). As the inferior conjunctival sac was in contact with the drug for the maximum time, it seems likely that the inferior part of the choroid received the highest drug concentration, thereby having the strongest effect on the choroid.

Atropine is a nonselective antagonist of the muscarinic acetylcholine receptor types M1, M2, M3, M4, and M5. Topical atropine is used as a cycloplegic, to temporarily paralyze the accommodation reflex, and also as a mydriatic agent, to dilate the pupils. There has been a relatively long time since atropine was used as an anti-myopia drug. In 1989, Yen *et al*.^17^ conducted the first randomized control trial on the use of atropine in childhood myopia, confirming that 1% atropine was effective in slowing the progression of myopia, and the effect of 1% atropine was better than that of 1% cyclopentolate. The latest study reported that 0.01% atropine now is the preferred option for controlling myopia progression than any other concentrations when considering side effects^13^. Unfortunately, the exact mechanism of atropine in slowing progression are still not known^14^,^15^. In the present study, the choroidal thickness at all measured locations significantly thickened after 1-week administration of 1% atropine gel, which may be a part of mechanism of preventing myopia progression in the use of atropine. Read and associates^16^ reported that children showing faster axial eye growth exhibited significantly less choroidal thickening over time compared with children showing slower axial eye growth. Besides, in an experimental study, Nickla and associates^18^ found that choroidal thickness could predict ocular growth rates in normal chick eyes: eyes with thinner choroids grew faster than those with thicker choroids. The aforementioned results further supported that there may be a potential role for the thicker choroid in the mechanisms inhibiting eye growth in childhood.

Atropine induces cycloplegia by paralyzing the ciliary muscles, whose action inhibits accommodation to allow accurate refraction in children. In China, children who are less than 10 years old are usually administered 1% atropine gel twice daily for a week or thrice daily for 3 successive days before refraction to reach cycloplegia. Compared to tropicamide (a shorter-acting cholinergic antagonist) or phenylephrine (an α-adrenergic agonist), 1% atropine gel when used twice daily for a week may be potent enough to reach and affect the choroid.

The exact mechanism underlying increased choroidal thickness following administration of topical 1% atropine, though elusive, could still be surmised from the findings of previous studies. Firstly, cycloplegia induced by atropine may be a very important factor that can increase the choroidal thickness, as some previous studies highlight an inverse relationship between accommodation and choroidal thickness^19^,^20^. It is well known that accommodation is highly efficient in children less than 10 years of age. During accommodation the force involved in the contraction of the ciliary muscle may be transmitted to the choroid, and this mechanical force can affect the choroidal thickness. Woodman *et al*.^19^ were first to observe a small amount of choroidal thinning during the accommodation task using Spectralis OCT. They postulated that the regional variation in the parafoveal thickness corresponds to the distribution of the nonvascular smooth muscle (NVSM) cells within the choroid indicating that these cells may regulate choroidal thickness during accommodation^20^. Chiang and associates^21^ found that subfoveal choroidal thickness significantly increased within 10 min of exposure to 2.00 D of monocular myopic defocus (reducing accommodation). Interestingly, Li *et al*.^22^ reported that accommodation decreased early after myopic refractive surgery with a concomitant increase in choroidal thickness, and the decrease in the amplitude of accommodation was the most significant factor associated with the increase of choroidal thickness at the fovea. Thus, in line with previous reports, blockade of accommodation by atropine may be an important mechanism underlying changes in choroidal thickness.

Secondly, antagonism of muscarinic receptors may be another possible factor affecting choroidal thickness. Alterations in the tone of nonvascular smooth muscle have been suggested to cause choroidal thickening after administration of antimuscarinic drugs in animals^1^. There is a rich autonomic vasoactive never supply to the choroid involving the activation of adrenergic and muscarinic receptors^2^. A previous study has found that electrical stimulation of the post-ganglionic axons (action of the parasympathetic innervation) from the chick ciliary ganglion leads to contraction of explant choroids, and this contraction can be blocked by atropine, showing that acetylcholine may affect contraction of at least some choroidal smooth muscle^23^. In addition, Nickla *et al*.^24^ have found that the choroid thickened significantly after double parasympathectomy in 4–5-week-old chicks. In another study by Nickla and associates^25^, it was observed that atropine inhibited the development of myopia in negative lens-wearing eyes, and also caused choroidal thickening in young chicks. It was proposed that the choroidal thickening reflected the loss of an excitatory input, probably cholinergic, to the choroidal nonvascular smooth muscle^23^, which may normally restrain thickening in opposition to osmotic forces that swell the lacunae^26^ and thicken the choroid^1^.

Given that the choroid contains abundant nonvascular smooth muscle, the contraction of these muscles might squeeze fluid out of the choroid, thereby thinning it and the contraction of nonvascular smooth muscle may be induced by acetylcholine^25^. However, because the nonvascular smooth muscle is not aligned perpendicular to the plane of the choroid, it is also possible that contraction of these muscles facilitates filling of the lacunae. Therefore, the contribution of nonvascular smooth muscle to the change in choroidal thickness is still speculative and needs further investigation.

Thirdly, considering the common molecular origin and high degree of homology between the muscarinic and adrenergic receptor families, cross-reactivity of some muscarinic antagonists with adrenergic receptors is likely^27^. For example, muscarinic toxin 3 (MT3, with high selectivity for the M_4_ receptor) has been shown to bind with high affinity to α-adrenergic receptors in mammals^28^. It is possible that such cross-reactivity may also be displayed by other muscarinic receptor antagonists including atropine. Further, previous studies have revealed that atropine, especially at high concentrations, had direct α-adrenoceptor blocking activity, which may account, at least in part, for the “atropine flush”^29^,^30^. In view of the above, such cross-reactivity could also affect the choroidal vasculature that is innervated by both sympathetic and parasympathetic nerves. Therefore, blockade of α-adrenoceptor in the choroidal vascular could also cause thickening of the choroid.

Finally, a non-adrenergic, non-cholinergic (NANC) dilator response which is potentiated by blockade of presynaptic muscarinic receptors may also be a plausible reason. Anticholinergic drugs such as atropine caused a vasodilation response in ocular blood vessels, potentially through their action on pre-junctional muscarinic receptors (possibly the M2 subtype), thus increasing the release of neural nitric oxide (NO) from vasodilator nerves^31^. In an ARVO annual meeting abstract, Carr *et al*.^32^ found that atropine could stimulate cyclic adenosine monophosphate (cAMP) synthesis in NO-ergic neurons, thereof producing abundant of NO, and NO is necessary for choroidal thickening^33^. Moreover, it is possible that atropine may indirectly affect the retina, by causing the release of dopamine or other neurotransmitters^34^, and it has been reported that dopamine can cause increase in choroidal thickness^35^.

To date, there are seven studies that report the effect of topical anticholinergic or adrenergic agents on the choroidal thickness. The details of these studies have been summarized in Table 1. Clearly, different results can be obtained even with the use of the same anticholinergic agents such as Mydrin-P, and tropicamide. We speculate that such discordance may be attributed to the difference in the race of participants, methods of instillation, bioactivities of anticholinergic agents^36^, and variations in individual responses to topical ocular eye drops. For example, the efficacy of all antimuscarinic agents is influenced by the amount of iris pigmentation. In addition, the amount of topical instillation that reaches the posterior is also an important factor that needs to be taken into account. There are three potential routes for penetration of topically applied ophthalmic drugs to the posterior segment: (1) the trans-vitreous route: trans-corneal diffusion followed by entry into vitreous and subsequent distribution to ocular tissues; (2) periocular route: diffusion around the sclera followed by trans-scleral absorption and (3) uvea-scleral route: trans-corneal diffusion followed by progression through the uvea-sclera^37^. Among these, the conjunctiva/scleral route is more important that it allows diffusion of drug into more posterior structures of the uveal tract. Atropine is a hydrophilic drug that can easily penetrate the sclera and reach the choroid.

It would have been ideal to study changes in different components of choroidal vasculature, similar to those reported in previous studies^38^,^39^,^40^,^41^; unfortunately, it was difficult to differentiate the various segmentations of choroidal vasculature in the present study. Therefore, we could not determine the exact reason for the thickening of choroid induced by atropine. This is a potentially interesting topic and deserves further explorations in the near future. Further, it would also be interesting to explore the proportionate change in the luminal and stromal areas of choroid before and after administration of atropine^42^.

The present study had some limitations that need to be considered. First, the sample size is relatively small and only children aged between 5 and 10 years were recruited. Therefore, the effect of atropine in the choroids of adults is unclear. Second, automated quantifying of choroidal thickness via software is yet not available commercially. Therefore, the choroidal thickness was manually measured using the built-in measuring tool. The wide range of 95% limitis of agreement in Fig. 2 and the large repeatability coefficients (more than 40 μm) indicated a large inter-observer variability in the present study. In order to reduce the error of the measurements, the choroidal thickness recorded by the two observers were averaged to obtain the final measurement at each point. Further research with larger sample sizes is needed to better elucidate the effect of atropine on the choroidal thickness. Third, we could not analyze retinal thickness following atropine administration as some of the children were too young to cooperate after OCT scan in the choroidal mode.

In conclusion, in the present study, we found that topical administration of 1% atropine gel can significantly increase choroidal thickness in young Chinese children, with different magnitude of change at different locations. However, the change in which segmentation of choroidal vasculature contributes to the thickening is still unclear and warrants further exploration using advanced OCT technologies.

## Subjects and Methods

This was a prospective comparative study. The Institutional Review Board of Nanjing Medical University Affiliated Wuxi Second Hospital approved the protocol. All participants gave their informed consent to participate in the clinical examination program, and our study was performed in accordance with the tenets of the Declaration of Helsinki.

### Subjects

Thirty-five healthy children were initially recruited into this comparative study between December 2015 and March 2016. Subjects with a history of any ophthalmic disease (except for refractive errors) were excluded. All subjects were screened for the presence of ocular diseases through a complete ophthalmologic examination, including fundus examination. Autorefraction was carried out with the Topcon autorefractor (KR-8900) to obtain the refractive states of participants after cycloplegia. A mean spherical equivalent (SE) was calculated as sphere power plus half the cylindrical power. The axial length was measured with the IOLMaster 500 (software version: 7.5.3.0084, Carl Zeiss Meditec, Dublin, CA). It is well known that the IOLMaster is simple to use and ideal for axial length assessment^43^, even in children^44^. Fifty-nine eyes of 30 subjects satisfying the inclusion criteria were finally assessed in the study. A single drop of 1% atropine gel was administered twice daily for a week. Because of the potential side effects of the drug, a careful history and assessment of the anterior chamber angle was made before use.

### Choroidal thickness measurement

All children underwent spectral-domain optical coherence tomography (SD-OCT) before and after one week of 1% atropine treatment that induced a continuous cycloplegia. Choroidal imaging was performed using a high-speed 840-nm-wavelength SD-OCT instrument (RTVue XR Avanti; Optovue, Inc, Fremont, California, USA). All OCT scans were performed by an operator (T.T.C) at approximately 0900 and 1100 hours both before and after cycloplegia, to avoid diurnal variations^45^,^46^. Fovea-centered, 6-mm horizontal and vertical high definition cross-line scans were taken with the chorioretinal line mode. Post-cycloplegia scans were obtained using the follow-up mode to obtain images from the previous location. All images underwent manual segmentation by two researchers in a blind-manner. RTVue manual measurement tools included calipers for delineating the boundaries of the choroid.

Choroidal thickness was determined as the perpendicular distance between the outermost edge of the hyper-reflective line of the retinal pigment epithelium and the sclerochoroidal border, as reported previously^47^. Choroidal thickness was measured at the subfovea and at 1.0 mm intervals (up to 3.0 mm) from the fovea at superior, inferior, nasal, and temporal locations. All parameters were measured by two independent observers. Choroidal thickness values recorded by the two observers were averaged to obtain the final measurement at each point. In case of a discrepancy in the measurement of choroidal thickness i.e. if the difference in choroidal thickness values recorded by the two observers exceeded by 20% of the mean of the two values, then the measurements were checked by another senior observer who gave the final adjudication, as reported in our previous study^47^.

### Measurement Reproducibility

To assess reliability of measurements of choroidal thcikness, interobserver reproducibility was evaluated using intraclass correlation coefficient (ICC)^48^. The ICC is an index of measurement reliability that ranges from 0 to 1, with values of the ICC of 0.81–1.00 indicating almost perfect agreement. Besides, Bland-Altman plots were performed to see if there was any proportional bias between measurements^49^. A 95% limitis of agreement (LOA) was defined as mean ± 1.96* standard deviation. In addition, the repeatability coefficient were also calculated for the inter-observer measurements according to the methods outlined by Bland and Altman. Specifically, the repeatability coefficient was defined as the standard deviation of the difference between the two repeated measurements multiplied by 1.96^49^.

### Statistical Analysis

All values of choroidal thickness are expressed as mean ± SD. Statistical analyses were performed using SPSS software (version 18.0, SPSS, Chicago, IL, USA). A one-sample Kolmogorov-Smirnov test was used to assess the normal distribution of continuous variables before a test of significance was applied. As a result, all of the continuous variables were normally distributed. Choroidal thickness values after a-week of topical 1% atropine gel treatment were compared to the baseline using paired Student’s *t*-test. The changes of choroidal thickness in the different meridian (temporal, nasal, superior and inferior) was analyzed using one way analysis of variance (ANOVA). *P* < 0.05 was considered statistically significant. All tests were two-tailed.

## Additional Information

**How to cite this article**: Zhang, Z. *et al*. The effect of topical atropine on the choroidal thickness of healthy children. *Sci. Rep.* **6**, 34936; doi: 10.1038/srep34936 (2016).

## Figures and Tables

**Table 1:** The summary of influence of different mydriatics in choroidal thickness.

Author	Year	Country	Sample size (eyes)	Mydriatics	Methods of instillations and OCT scans	demographics and ocular parameters				OCT manufacturers	Results
						Age (year)	Gender (F/M)	SE (diopters)	AXL (mm)		
Kim	2012	Korea	58	Mydrin-P*	a single drop of Mydrin-P, three times at 5 min intervals. OCT scans were performed when pupil size>6 mm.	33.24 ± 14.25	12/17	−2.16 ± 2.01	24.27 ± 1.12	Heidelberg	No significant influence
Mwanza	2013	USA	17	Tropicamide	a single drop of tropicamide 1%, OCT scans were performed at 20–30 min after pupil dilation, with the option of a second drop if the first failed to dilate the pupil enough.	53.50 ± 13.90	—	—	23.70 ± 1.30	Heidelberg	No significant influence
			40#			69.60 ± 13.10	—	—	23.90 ± 1.50		
Hao	2013	China	116	Mydrin-P*	a single drop of Mydrin-P, four times at 10 min intervals. OCT scans were performed at 30 min after the last instillation when pupil size > 8 mm.	12.20 ± 2.50	30/28	−2.4 ± 1.17	—	Heidelberg	Thinner
Kara	2014	Turkey	30	Tropicamide	a drop of 1% tropicamide thrice at 5 min intervals, EDI-OCT measurements were performed at 45 min after the last instillation.	33 ± 10	16/14	0.06 ± 0.85	23.38 ± 1.23	Heidelberg	Thinner
			30	Phenylephrine	a drop of 2.5% phenylephrine thrice at 5 min intervals, EDI-OCT measurements were performed at 45 min after the last instillation.	39 ± 12	19/11	0.11 ± 0.36	23.47 ± 1.05		
Sander	2014	Australia	14	Homatropine	a drop of 2% homatropine hydrobromide, ocular measurements taken 30 and 60 min following the last instillation.	27.9 ± 4.0	5/9	−0.62 ± 1.42	—	Optopol	Thicker
			14	Phenylephrine	a drop of 2.5% phenylephrine hydrochloride, ocular measurements taken 30 and 60 min following the instillation.	27.9 ± 4.0	5/9	−0.62 ± 1.42	—		No significant influence
Yuvacı	2015	Turkey	29	Tropicamide	a single drop of tropicamide 1% 3 times at 5 min intervals, OCT scans were performed at 50 min after instillation.	32.9 ± 1.4	12/17	−0.55 ± 1.49	23.4 ± 0.63	Heidelberg	Thinner
			29	Phenylephrine	a single drop of 2.5% phenylephrine 3 times at 5 min intervals, OCT scans were performed at 50 min after the last instillation.	31.8 ± 1.5	13/16	−0.73 ± 1.60	23.7 ± 0.81		
			32	Cyclopentolate	a single drop of cyclopentolate 1% 3 times at 5 min intervals, OCT scans were performed at 50 min after the last instillation.	28.9 ± 1.6	18/14	0.01 ± 2.24	23.3 ± 0.88		
Öner	2016	Turkey	37	Tropicamide	a single drop of tropicamide 1% 3 times at 10 min intervals, OCT scans were performed at 40 min after the last instillation.	41.4 ± 14.3	19/18	0.36 ± 0.8	—	Carl Zeiss	No significant influence
			37	Cyclopentolate	a single drop of cyclopentolate 1% 3 times at 10 min intervals, OCT scans were performed at 40 min after the last instillation.	43.8 ± 16.5	20/17	0.68 ± 1.4	—		Thicker

^*^Tropicamide 5 mg/mL and phenylephrine 5 mg/mL, Santen Pharmaceuticals, Japan.

^#^Including 20 eyes with normal tension glaucoma and 20 eyes with primary open-angle glaucoma, respectively.

**Table 2:** Changes of choroidal thickness before and after administration of 1% atropine gel.

Location	Before	After	Difference	*P*
Fovea	287.03 ± 65.76	302.52 ± 69.94	15.48 ± 16.13	<0.001
Nasal 1.0 mm	250.80 ± 59.37	268.20 ± 61.44	17.40 ± 15.93	<0.001
Nasal 2.0 mm	200.40 ± 53.69	212.40 ± 53.23	12.00 ± 16.89	0.001
Nasal 3.0 mm	160.67 ± 38.97	164.83 ± 39.04	4.17 ± 10.49	0.038
Temporal 1.0 mm	283.63 ± 68.30	304.17 ± 76.93	20.53 ± 25.73	<0.001
Temporal 2.0 mm	281.17 ± 59.90	298.93 ± 68.19	17.77 ± 23.27	<0.001
Temporal 3.0 mm	272.63 ± 52.89	292.90 ± 61.53	20.27 ± 23.85	<0.001
Inferior 1.0 mm	289.67 ± 68.53	305.70 ± 66.85	16.03 ± 23.22	0.001
Inferior 2.0 mm	281.23 ± 63.04	306.20 ± 64.37	24.97 ± 37.59	0.001
Inferior 3.0 mm	274.70 ± 69.31	295.03 ± 70.61	20.33 ± 28.41	<0.001
Superior 1.0 mm	282.27 ± 63.22	294.70 ± 67.61	12.43 ± 26.13	0.014
Superior 2.0 mm	271.87 ± 57.68	288.13 ± 61.02	16.27 ± 19.76	<0.001
Superior 3.0 mm	253.60 ± 53.74	265.87 ± 59.95	12.27 ± 19.06	0.001

**Table 3:** Paired mean difference, repeatability coefficient, and Intraclass correlation for choroidal thickness measurement between the two observasers before and after cycloplegia.

	paired mean difference, μm (95% LOA)	repeatability coefficient, μm (95% CI)	ICC (95% CI)	***P*** value
Before cycloplegia	2.61 (−43.09, 48.31)	46.58 (45.55, 47.32)	0.952 (0.933, 0.974)	<0.001
After cycloplegia	1.47 (−38.77, 41.71)	40.96 (39.58, 41.14)	0.955 (0.941, 0.976)	<0.001

LOA, limits of agreement; ICC, intraclass correlation coefficient; CI, confidence interval
